# Synthesis, docking and acetylcholinesterase inhibitory assessment of 2-(2-(4-Benzylpiperazin-1-yl)ethyl)isoindoline-1,3-dione derivatives with potential anti-Alzheimer effects

**DOI:** 10.1186/2008-2231-21-47

**Published:** 2013-06-07

**Authors:** Ahmad Mohammadi-Farani, Aram Ahmadi, Hamid Nadri, Alireza Aliabadi

**Affiliations:** 1 Department of Pharmacology, Toxicology and Medical Services, Faculty of Pharmacy, Kermanshah University of Medical Sciences, Kermanshah, Iran; 2 Students Research Committee, Kermanshah University of Medical Sciences, Kermanshah, Iran; 3 Neurobiomedical Research Center, Shahid Sadoughi University of Medical Sciences, Yazd, Iran; 4 Department of Medicinal Chemistry, Faculty of Pharmacy, Kermanshah University of Medical Sciences, Kermanshah, Iran

**Keywords:** Synthesis, Phthalimide, Acetylcholinesterase (AChE), Anti-Alzheimer, Ellman test, Docking

## Abstract

**Background:**

Alzheimer’s disease (AD) as neurodegenerative disorder, is the most common form of dementia accounting for about 50-60% of the overall cases of dementia among persons over 65 years of age. Low acetylcholine (ACh) concentration in hippocampus and cortex areas of the brain is one of the main reasons for this disease. In recent years, acetylcholinesterase (AChE) inhibitors like donepezil with prevention of acetylcholine hydrolysis can enhance the duration of action of acetylcholine in synaptic cleft and improve the dementia associated with Alzheimer’s disease.

**Results:**

Design, synthesis and assessment of anticholinesterase activity of 2-(2-(4-Benzylpiperazin-1-yl)ethyl)isoindoline-1,3-dione derivatives showed prepared compounds can function as potential acetylcholinesterase inhibitor. Among 12 synthesized derivatives, compound 4a with *ortho* chlorine moiety as electron withdrawing group exhibited the highest potency in these series (IC_50_ = 0.91 ± 0.045 μM) compared to donepezil (IC_50_ = 0.14 ± 0.03 μM). The results of the enzyme inhibition test (Ellman test) showed that electron withdrawing groups like Cl, F and NO_2_ can render the best effect at position *ortho* and *para* of the phenyl ring. But compound 4g with methoxy group at position 3(*meta*) afforded a favorable potency (IC_50_ = 5.5 ± 0.7 μM). Furthermore, docking study confirmed a same binding mode like donepezil for compound 4a.

**Conclusions:**

Synthesized compounds 4a-4l could be proposed as potential anticholinesterase agents.

## Background

Alzheimer’s disease (AD), is described by Dr. Alois Alzheimer in 1907 as a neurodegenerative disorder. AD as a disease of the central nervous system (CNS) characterized especially by premature senile mental deterioration. AD patients exhibit a significant decrease in cognitive ability and severe behavioral and psychological abnormalities such as irritability, anxiety and depression [1]. AD, one of the most diffuse neurodegenerative pathology among the elderly, causes a progressive impairment in functional performances and continuous reduction in cognitive activities and memory [2-7]. AD is the most common form of dementia accounting for about 50-60% of the overall cases of dementia among persons over 65 years of age, is a neurodegenerative alteration characterized by a low acetylcholine (ACh) in hippocampus and cortex [8]. AD is said to be the leading cause of dementia in elderly patients. Nowadays, with increase of the elderly population, the prevalence of AD is likely to increase. AD persons exert a decrease in mental functions and performances and consequently rendering them incapacitated and unable to perform normal daily activities. Elderly persons are the most common individuals afflicted with this disease. Unfortunately, the true nature or cause of the disease is still unknown and therefore, the development of effective anti-alzheimer is one of the encouraging area in current medicinal chemistry researches [1].

Over two decades ago, several autopsy studies inside hippocampus revealed that the levels of the neurotransmitter acetylcholine in patients with Alzheimer’s disease are importantly decreased [9]. Currently, the loss of cholinergic function is the only evidentiary finding responsible for cognitive decline. Hence, therapeutical development has focused on this theory. The loss of the basal forebrain cholinergic system is one of the most significant aspects of neurodegeneration in the brains of AD patients, and it is thought to play a central role in producing cognitive impairments [1,2,10,11]. Alzheimer’s disease leads to a progressive decline of the cognitive function, executive function losses, memory deficits, and eventually to incapacitating dementia before death. In order to improve cholinergic neurotransmission, different strategies have been investigated including the increase of synthesis or pre-synaptic release of ACh, the stimulation of cholinergic post-synaptic muscarinic and nicotinic receptors, and the reduction of ACh synaptic degradation using AChE inhibitors (e.g., AChEIs or anticholinesterase agents) [12-19].

**Figure 1:**
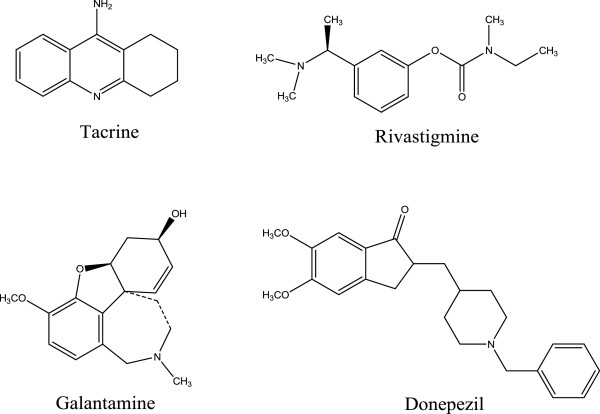
Structures of acetycholinesterase inhibitors in the market.

Acetylcholinesterase inhibitors have beneficial effects on cognitive, functional, and behavioral symptoms of AD. A recent review by the Quality Standard Subcommittee of the American Academy of Neurology investigated important issues in the management of dementia. The reviewers concluded that despite the small average degree of benefit of treatment with acetylcholinesterase inhibitors they should be the first-line treatment in patients with mild to moderate AD [4]. Four cholinesterase inhibitors tacrine, donepezil, rivastigmine, and galantamine are approved by the US Food and Drug Administration (FDA) and are currently in the market (Figure 1) [5].

**Figure 2:**
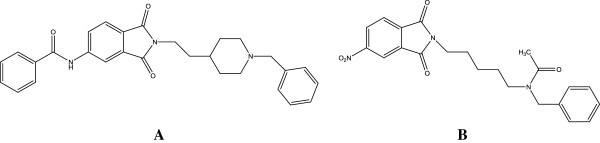
Two structures of phthalimide based anticholinesterase.

According to the several reports about the efficacy of phthalimide derivatives in inhibition of AChE, [20-27] (Figure 2) in the current project, we focused on the design and synthesis of new anticholinesterase agents with phthalimide-based structure to reach more active analogs towards inhibition of AChE. Besides, we hope the new analogs to render lower side effects. Improving the pharmacokinetic properties of the designed compounds could be another aim of this study. In fact, phthalimide based compounds have similar pharmacophoric portions like indanone ring of the donepezil and are able to act as peripheral binding site inhibitor of AChE. We also investigated *in silico* binding mode of proposed ligands into the acetylcholinesterase enzyme in comparison with donepezil as reference drug by docking procedure.

## Methods

### Preliminary design

**Figure 3:**
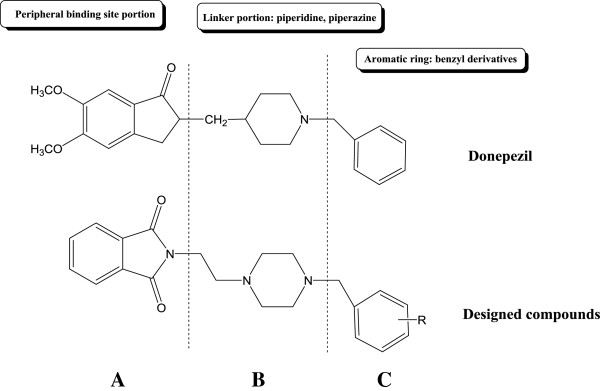
Design of new donepezil-like analogs based on the phthalimide structure.

In the recent years, there are several reports about the potentiality of phthalimide derivatives as potent anticholinesterase agents. In fact, the phthalimide-based compounds can function as inhibitor of the peripheral binding site of AChE enzyme [20-27]. On the other hands, it is well-known in medicinal chemistry that piperazine ring can act as good bioisosteric replacement for piperidine moiety. According to the Figure 3, we designed a new series of donepezil-like analogues with replacement of the indanone and piperidine rings of the donepezil with phthalimide (or isoindoline-1,3-dione) group and piperazine ring respectively. As illustrated in Figure 3, there are three distinct and critical portions in the structure of donepezil. Part A or peripheral binding site portion is necessary for binding to the AChE enzyme. The phenyl ring of the indanone moiety of donepezil participates in π-π stacking interaction with indole ring of Trp 279 in the active site of AChE. Part B is a linker region as well as an interacting part with Phe 330 in the active site of the AChE. According to the structure activity relationship (SAR) of donepezil-like analogs, the nitrogen atom of the piperidine ring is necessary for binding to the active site of the enzyme and removing of the nitrogen can be detrimental for anticholinesterase activity of the ligand. In fact, the charged nitrogen of the piperidine ring makes a cation-π interaction with Phe 330. Pat C or benzyl moiety unit plays its role through a π-π stacking interaction with indole ring of the Trp 84 [16]. Hence, in the designed compounds (Figure 3), the considering of these three pivotal portions of the donepezil was carried out and subsequently the synthesis of proposed compounds was done.

### Chemistry

All chemical substances consisting starter materials, reagents and solvents were purchased from the commercial supplier like Merck and Sigma-Aldrich companies. The purity of the prepared compounds was proved by thin layer chromatography (TLC) using various solvents of different polarities. Merck silica gel 60 F_254_ plates were used for analytical TLC. Column chromatography was applied on Merck silica gel (70–230 mesh) for purification of intermediate and final compounds. ^1^H-NMR spectra were recorded using a Bruker 400 MHz spectrometer in deutrated solvents, and chemical shifts are expressed as δ (ppm) with tetramethylsilane (TMS) as internal standard. The IR spectra were obtained on a Shimadzu 470 spectrophotometer using potassium bromide (KBr) disks. Melting points were determined using Electrothermal 9001 elemental analyzer apparatus and are uncorrected. The mass spectra were run on a Finigan TSQ-70 spectrometer (Finigan, USA) at 70 eV.

#### *Synthesis of 2-(2-(piperazin-1-yl)ethyl)isoindoline-1,3-dione***(3)**

In a flask 3 g (20 mmol) of Phthalic anhydride, 2.6 ml (20 mmol) *N*-amnioethylpiperazine and 2.9 ml (20 mmol) triethylamine (Et_3_N) were mixed in 40 ml of toluene solvent. The reaction mixture was refluxed for 24 hours and the termination of reaction and formation of the desired product was confirmed by thin layer chromatography. The discoloration of the reaction medium and formation of a yellow precipitate was also an indicator of the progress of the reaction. Then, toluene was evaporated under reduced pressure using rotary evaporator apparatus and the obtained yellow viscose and oily residue was washed several times by ethyl acetate (EtOAc) and diethyl ether (Et_2_O) [28].

^1^H NMR (CDCl_3_, 400 MHz) δ (ppm): 2.37 (m, 4H, Piperazine), 2.54 (m, 4H, Piperazine), 3.22 (t, 2H, phthalimide-CH_2_-CH_2_-piperazine), 3.44 (t, 2H, phthalimide-CH_2_-CH_2_-piperazine), 4.73 (NH, Piperazine), 7.35-7.85 (m, 4H, Phthalimide). IR (KBr, cm^-1^) ῡ: 3380, 3330, 3157, 3111, 2924, 1730, 1681, 1521, 1489, 1458, 1328, 1303, 1186, 1143, 1035, 910, 750, 710. MS (*m/z*, %): 259 (M^+^, 10), 224 (30), 174 (30), 160 (60), 149 (85), 99 (100), 70 (70), 57 (65), 41 (40).

#### *General procedure for synthesis of compounds* 4a-4l

In a flat-bottom flask equimolar quantities of compound 3 and appropriate derivative of benzyl chloride were added together in dichloromethane (CH_2_Cl_2_) solvent. The reaction mixture was stirred in room temperature overnight. Then, dichloromethane was evaporated under reduced pressure and the afforded residue was washed by diethyl ether and *n*-hexane. Methanol containing Hydrochloric acid gas was added to the residue to form the related hydrochloride salt of the product [29].

#### *2-(2-(4-(2-Chlorobenzyl)piperazin-1-yl)ethyl)isoindoline-1,3-dione* (4a)

^1^H NMR (DMSO-d_6_, 400 MHz) δ (ppm): 2.36 (m, 8H, Piperazine), 3.33 (t, 2H, CH_2_-piperazine), 3.35 (s, 2H, -CH_2_-2-Chlorophenyl), 3.61 (t, 2H, -CH_2_-Phthalimide), 7.25-7.32 (m, 2H, 2-Chlorophenyl), 7.34-7.44 (m, 2H, 2-Chlorophenyl), 7.46-7.87 (m, 4H, Phthalimide). MS (*m/z*, %): 385 (M^+^+2, 2), 383 (M^+^, 5), 225 (50), 223 (100), 160 (50), 125 (95), 89 (15), 70 (20). IR (KBr, cm^-1^) ῡ: 3460, 3051, 2943, 2800, 2762, 1766, 1465, 1438, 1396, 1357, 1300, 1192, 1161, 1107, 1041, 921, 759, 717, 698.

#### *2-(2-(4-(3-Chlorobenzyl)piperazin-1-yl)ethyl)isoindoline-1,3-dione* (4b)

^1^H NMR (DMSO-d_6_, 400 MHz) δ (ppm): 2.34 (m, 4H, Piperazine), 3.35 (t, 2H, -CH_2_-piperazine), 3.51 (s, 2H, -CH_2_-phenyl), 3.7 (t, 2H, -CH_2_-phthalimide), 7.27-7.32 (m, H_2_, H_5_, 3-Chlorophenyl), 7.41 (d, 1H, *J* = 8 Hz, H_6_-3-Chlorophenyl), 7.45 (d, *J* = 8 Hz, H_4_-3-Chlorophenyl), 7.85 (m, 2H, H_5_,H_6_-phthalimide), 7.88 (m, 2H, H_4_,H_7_-phthalimide). IR (KBr, cm^-1^) ῡ: 3160, 3113, 2924, 1710, 1681, 1533, 1521, 1489, 1458, 1327, 1303, 1186, 1143, 1037, 715.

#### *2-(2-(4-(4-Chlorobenzyl)piperazin-1-yl)ethyl)isoindoline-1,3-dione* (4c)

^1^H NMR (CDCl_3_, 400 MHz) δ (ppm): 2.2 (t, 2H, -N-CH_2_-CH_2_-NH-, piperazine), 2.5 (t, 2H, -N-CH_2_-CH_2_-NH-, Piperazine), 2.63 (t, 2H, phthalimide-CH_2_-CH_2_-piperazine), 3.42 (s, 2H, -CH_2_-phenyl), 3.8 (t, 2H, phthalimide-CH_2_-CH_2_-piperazine), 7.23 (d, 2H, *J* = 8 Hz, H_2,6_-4-chlorophenyl), 7.30 (d, 2H, *J* = 8 Hz, H_3,5_-4-chlorophenyl phenyl), 7.76 (m, 4H, Phthalimide). IR (KBr, cm^-1^) ῡ: 3400, 3380, 3135, 3111, 2927, 2812, 1703, 1681, 1533, 1521, 1489, 1456, 1328, 1305, 1186, 1143, 1035, 721, 707. MS (*m/z*, %): 384 (M^+^ +1, 20), 383 (M^+^, 18), 280 (20), 223 (100), 167 (30), 149 (90), 125 (90).

#### *2-(2-(4-(2-Fluorobenzyl)piperazin-1-yl)ethyl)isoindoline-1,3-dione* (4d)

^1^H NMR (DMSO-d_6_, 400 MHz) δ (ppm): 2.4 (m, 4H, piperazine), 3.1 (m, 4H, aliphatic), 3.8 (s, 2H, -CH_2_-phenyl), 7.35 (m, 4H, 2-Fluorophenyl), 7.88 (m, 4H, Phthalimide). IR (KBr, cm^-1^) ῡ: 3429, 2993, 2924, 2854, 1774, 1716, 1396, 1068, 1010, 721. MS (*m/z*, %): 368 (M^+^+1, 25), 313 (40), 285 (35), 257 (40), 236 (100), 152 (40), 111 (60), 97 (95), 83 (90), 69 (90), 57 (85), 43 (50).

#### *2-(2-(4-(3-Fluorobenzyl)piperazin-1-yl)ethyl)isoindoline-1,3-dione* (4e)

^1^H NMR (DMSO-d_6_, 400 MHz) δ (ppm): 3.37-3.87 (m, aliphatic), 3.99 (s, 2H, -CH_2_-phenyl), 7.29 (d, 1H, *J* = 8 Hz, H_2_-3-Fluorophenyl), 7.49 (m, 1H, H_6_-3-Fluorophenyl), 7.66 (d, 1H, *J* = 8 Hz, H_5_-3-Fluorophenyl), 7.68 (d, 1H, *J* = 8 Hz, H_3_-3-Fluorophenyl), 7.86-7.88 (m, 4H, phthalimide). IR (KBr, cm^-1^) ῡ: 3371, 2993, 2958, 2924, 2854, 1774, 1712, 1546, 1462, 1404, 1056, 972, 721.

#### *2-(2-(4-(4-Fluorobenzyl)piperazin-1-yl)ethyl)isoindoline-1,3-dione* (4f)

^1^H NMR (DMSO-d_6_, 400 MHz) δ (ppm): 3.99 (m, 12H, aliphatic), 3.96 (s, -CH_2_-phenyl), 3.99 (t, 2H, -CH_2_-phthalimide), 7.3 (t, 2H, *J* = 8 Hz, 4-Fluorophenyl), 7.58-7.73 (m, 4-Fluorophenyl), 7.83-7.88 (m, 4H, Phthalimide). IR (KBr, cm^-1^) ῡ: 3367, 2997, 2854, 1774, 1716, 1604, 1512, 1462, 1400, 1056, 975, 721.

#### *2-(2-(4-(3-Methoxybenzyl)piperazin-1-yl)ethyl)isoindoline-1,3-dione* (4g)

^1^H NMR (DMSO-d_6_, 400 MHz) δ (ppm): 3.2-3.66 (m, aliphatic), 3.79 (s, 3H, -OCH_3_), 3.97 (s, 2H, -CH_2_-phenyl), 7.1-7.36 (m, 4H, 4-Methoxyphenyl), 7.58-7.66 (m, 4H, Phthalimide). IR (KBr, cm^-1^) ῡ: 3371, 2993, 1774, 1716, 1612, 1462, 1435, 1400, 1381, 1269, 1056, 972, 906, 798, 721. MS (*m/z*, %): 379 (M^+^, 15), 219 (100), 160 (30), 121 (80), 91 (20).

#### *2-(2-(4-(4-Methoxybenzyl)piperazin-1-yl)ethyl)isoindoline-1,3-dione* (4h)

^1^H NMR (DMSO-d_6_, 400 MHz) δ (ppm): 3.1-3.78 (m, aliphatic), 3.78 (s, 3H, -OCH_3_), 4.4 (s, 2H, -CH_2_-phenyl), 7.1 (dd, 4H, *J* = 8 Hz, 4-Methoxyphenyl), 7.43-7.58 (m, 4H, Phthalimide). IR (KBr, cm^-1^) ῡ: 3429, 2978, 2935, 1778, 1716, 1612, 1516, 1462, 1435, 1400, 1235, 1184, 1072, 1029, 802, 725.

#### *2-(2-(4-(2-Nitrobenzyl)piperazin-1-yl)ethyl)isoindoline-1,3-dione* (4i)

^1^H NMR (DMSO-d_6_, 400 MHz) δ (ppm): 2.25-2.35 (m, 8H, Piperazine), 3.35 (t, 2H, -CH_2_-piperazine), 3.66 (s, 2H, CH_2_-phenyl), 3.68 (t, 2H, -CH_2_-phthalimide), 7.4-7.66 (m, 4H, 2-Nitrophenyl), 7.83-7.89 (m, 4H, Phthalimide). IR (KBr, cm^-1^) ῡ: 3155, 3111, 2956, 2922, 1681, 1521, 1489, 1458, 1327, 1303, 1188, 1141, 1035, 740, 717, 705. MS (*m/z*, %): 394 (M^+^, 3), 377 (15), 259 (30), 235 (40), 234 (100), 200 (50), 160 (70), 130 (30), 99 (55), 78 (30) 56 (15).

#### *2-(2-(4-(3-Nitrobenzyl)piperazin-1-yl)ethyl)isoindoline-1,3-dione* (4j)

^1^H NMR (DMSO-d_6_, 400 MHz) δ (ppm): 2.51 (m, 4H, Piperazine), 3.53 (t, 2H, -CH_2_-piperazine), 3.79 (s, -CH_2_-phenyl), 3.85 (t, 2H, -CH_2_-phthalimide), 7.6-7.66 (m, 2H, H_4,6_-3-Nitrophenyl), 7.65 (s, 1H, H_2_-3-Nitrophenyl), 7.75 (t, *J* = 8 Hz, 1H, H_5_-3-Nitrophenyl), 7.85 (m, 2H, H_5,6_-phthalimide), 7.88 (m, 2H, H_4,7_-phthalimide). IR (KBr, cm^-1^) ῡ: 3425, 2924, 2854, 1774, 1716, 1531, 1435, 1400, 1350, 1060, 806, 721.

#### *2-(2-(4-(4-Nitrobenzyl)piperazin-1-yl)ethyl)isoindoline-1,3-dione* (4k)

^1^H NMR (DMSO-d_6_, 400 MHz) δ (ppm): 2.3 (m, 8H, Piperazine), 3.33 (t, 2H, phthalimide-CH_2_-CH_2_-piperazine), 3.56 (s, 2H, -CH_2_-phenyl), 3.69 (t, 2H, phthalimide-CH_2_-CH_2_-piperazine), 7.57 (d, 2H, *J* = 8 Hz, H_2,6_-4-Nitrophenyl), 7.83-7.88 (m, 4H, Phthalimide), 8.19 (d, 2H, *J* = 8 Hz, H_3,5_-4-Nitrophenyl). IR (KBr, cm^-1^) ῡ: 3157, 3111, 2924, 1775, 1681, 1519, 1489, 1458, 1330, 1303, 1186, 1143, 1037, 750, 710. MS (*m/z*, %): 395 (M^+^+1, 10), 394 (M^+^, 35), 235 (85), 234 (100), 191 (60), 174 (30), 160 (85), 90 (25), 70 (40).

#### *2-(2-(4-Benzylpiperazin-1-yl)ethyl)isoindoline-1,3-dione* (4l)

^1^H NMR (DMSO-d_6_, 400 MHz) δ (ppm): 2.51 (m, 4H, Piperazine), 3.55 (m, 8H, aliphatic), 3.99 (s, 2H, -CH_2_-phenyl), 7.45-7.68 (m, 4H, phenyl), 7.85 (m, 4H, Phthalimide). IR (KBr, cm^-1^) ῡ: 3414, 2997, 2924, 1774, 1712, 1635, 1431, 1396, 1064, 721. MS (*m/z*, %): 349 (10), 234 (20), 189 (100), 160 (35), 91 (90).

### Anticholinesterase activity assay (Ellman test)

Lyophilized powder of acetylcholinesterase from electric eel source (AChE, E.C. 3.1.1.7, Type V-S, 1000 unit) was purchased from Sigma-Aldrich (Steinheim, Germany). 5,5’-Dithiobis-(2-nitrobenzoic acid), potassium dihydrogen phosphate, dipotassium hydrogen phosphate, potassium hydroxide, sodium hydrogen carbonate, and acetylthiocholine iodide were purchased from Fluka (Buchs, Switzerland). Compounds 4a-4l were dissolved in a mixture of 20 ml distilled water and 5 ml methanol and then diluted in 0.1 M KH_2_PO_4_/K_2_HPO_4_ buffer (pH 8.0) to afford a final concentration range. The Ellman test was carried out for assessment of the anticholinesterase activity of intended compounds *in vitro*. Prior to use, all solutions were adjusted to 25°C. To achieve 20-80% inhibition of AChE activity five different concentrations of each compound were tested. The assay solution consisted of a 0.1 M potassium phosphate buffer pH 8.0, with the addition of 0.01 M 5,50-dithio-bis(2-nitrobenzoic acid), 2.5 unit/mL of enzyme solution (AChE, E.C. 3.1.1.7, Type V-S, lyophilized powder, from electric eel) (Sigma Chemical). Compounds 4a-4l were added to the assay solution and preincubated at 25°C with the enzyme for 15 min followed by adding 0.075 M substrate (acetylthiocholine iodide). After rapid and immediate mixing the change of absorption was measured at 412 nm. In order to justify non enzymatic reaction assays were carried out with a blank containing all components except AChE.

The blank reading contained 3 ml buffer, 200 μl water, 100 μl DTNB and 20 μl substrate. The reaction rates were calculated, and the percent inhibition of test compounds was determined. Each concentration was analyzed in triplicate, and IC_50_ values were determined graphically from inhibition curves (log inhibitor concentration vs percent of inhibition). Spectrophotometric measurements were performed on a Cecil BioAquarius CE 7250 Double Beam Spectrophotometer [30].

### Docking

**Figure 4:**
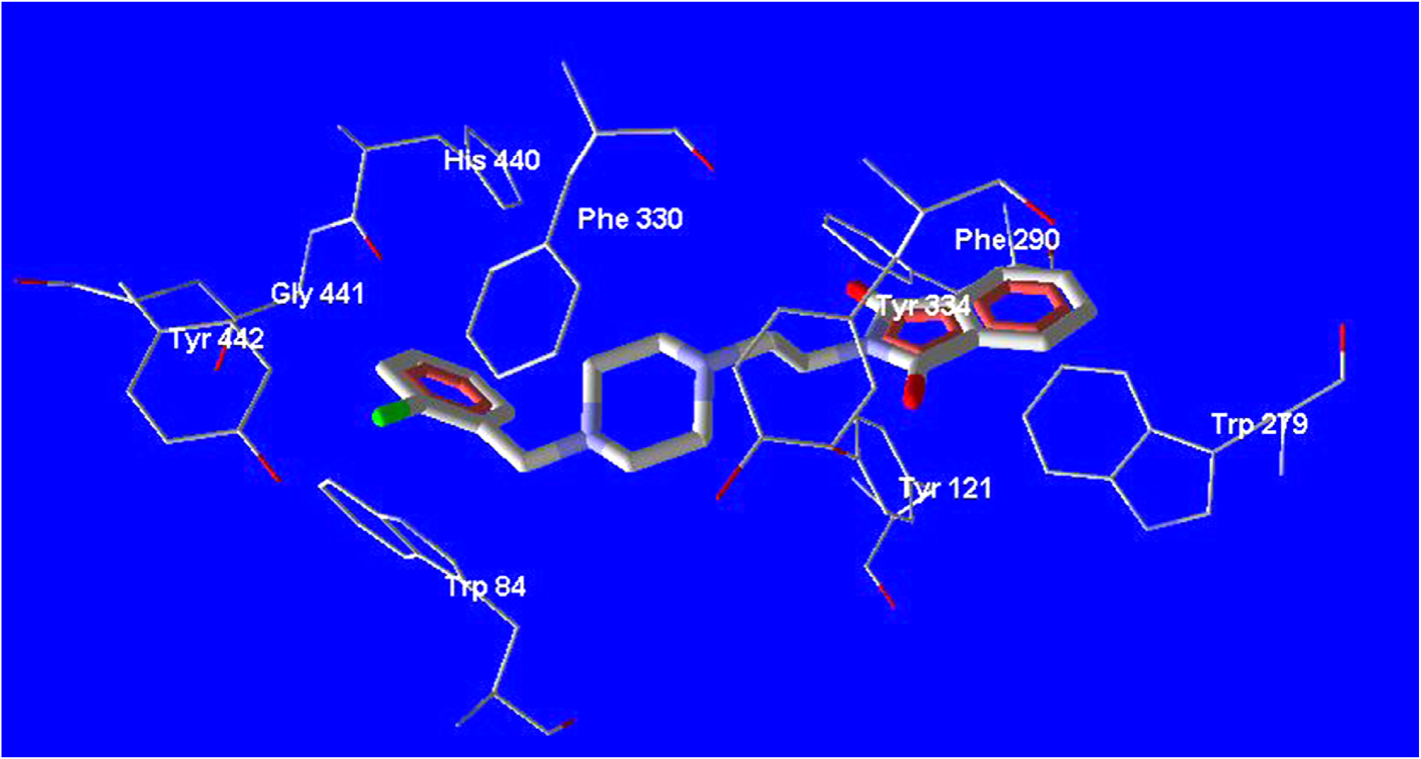
**Docked molecule of compound 4a in the active site of AChE (PDB code: 1EVE).** The principal amino acids (Trp 279, Phe 330, Trp 84) are evident in the nearby of the docked molecule.

**Figure 5:**
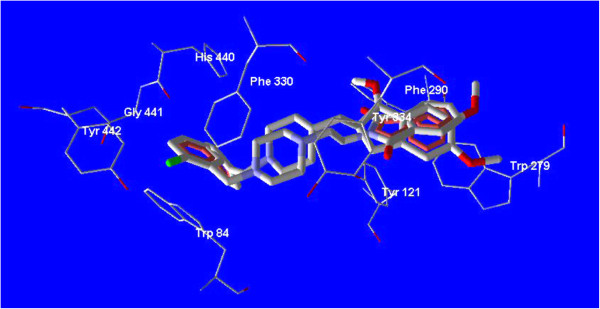
**Superimposed structure of compound 4a with donepezil in the active site of AChE.** A similar binding mode and interaction like donepezil are observed for compound 4a.

ArgusLab 4.0 software was applied to perform molecular docking studies [31,32]. All intended ligands were constructed in arguslab workspace and all ligands 4a-4l were energy minimized by AM1 as semiemperical method. The pdb file of acetylcholinesterase enzyme in complex with donepezil (pdb code: 1EVE) was downloaded from brookhaven protein databank [33]. The geometry optimization of structure of acetylcholinesterase enzyme was performed using universal force field (UFF) as a molecular mechanic method. The docking process was done for all ligands in the workspace of ArgusLab 4 software after defining the related groups for each ligand and also for related protein. The binding location of donepezil was defined as binding site for finding the best pose and conformation for all ligands. Binding mode and related interactions of all ligands were explored in Molegro molecular viewer software (Figure 4, Figure 5) [34].

## Results and discussion

### Chemistry

**Scheme 1:**
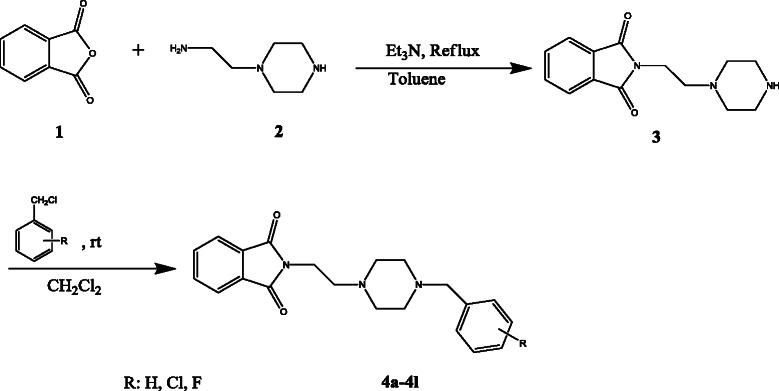
Synthetic pathway of compounds 4a-4l.

As summarized in the Scheme 1, all intermediate and final compounds 3 and 4a-4l were synthesized with an acceptable to high yield (ranging 61–96%) as listed in Table 1. Compound 3 was obtained through a Gabriel synthetic reaction. Reaction of phthalic anhydride with *N*-aminoethylpiperazine afforded the compound 3 with 61% yield. The reaction was carried out under reflux conditions in toluene solvent. Washing of yellowish oily product was done by ethyl acetate and *n*-hexane. A yellow powder was obtained after trituration. Obtained product was used for synthesis of 4a-4l derivatives. Room temperature stirring of equimolar quantities of compound **3** with appropriate benzyl chloride derivatives afforded 4a-4l derivatives. Dichloromethane was used as solvent and overnight stirring is necessary for achieving an acceptable yield. After evaporation, an oily residue was obtained. Methanolic hydrochloric acid was applied to form the corresponding hydrochloride salt of each derivative. For each compound 2 ml of methanolic solvent was added to the obtained residue and stirred at room temperature for 5–10 minutes. The formed precipitate was filtered, dried and collected.

**Table 1:** Properties of synthesized compounds

**Compounds**	**R**	**Closed formula**	**MW (g/mol)**	**mp (°C)**	**Yield (%)**	**Appearance**
3	-	C_14_H_17_N_3_O_2_	259	105-109	61	Yellow
4a	2-Cl	C_21_H_22_ Cl N_3_O_2_	383	105-107	81	Creamy
4b	3-Cl	C_21_H_22_ Cl N_3_O_2_	383	100-103	74	Creamy
4c	4-Cl	C_21_H_22_ Cl N_3_O_2_	383	122	78	Creamy
4d	2-F	C_21_H_22_FN_3_O_2_	367	226	96	Yellow
4e	3-F	C_21_H_22_FN_3_O_2_	367	225	77	Creamy
4f	4-F	C_21_H_22_FN_3_O_2_	367	293-296	77	Yellow
4g	3-OCH_3_	C_22_H_25_N_3_O_3_	379	259	82	Yellow
4h	4-OCH_3_	C_22_H_25_N_3_O_3_	379	265	65	Yellow
4i	2-NO_2_	C_21_H_22_N_4_O_4_	394	118-120	78	Orange
4j	3-NO_2_	C_21_H_22_N_4_O_4_	394	286	92	Orange
4k	4-NO_2_	C_21_H_22_N_4_O_4_	394	162	80	Creamy
4l	H	C_21_H_23_N_3_O	349	231	92	Orange

Different types of substituent consisted of three electron withdrawing substituents (Cl, F, NO_2_) and also an electron donating substituent (−OCH_3_) were synthesized to investigate the electronic effects of various moieties. Furthermore, compound 4l was also synthesized without any group on the phenyl ring to explore the effect of the presence of the substitution on this ring.

Melting points of intermediate and final compounds were obtained using capillary tubes by electrothermal melting point analyzer. A range in melting point was recorded for compounds 3, 4a, 4b, 4f and 4i. In other cases a sharp point was detected and presented in Table 1. ^1^H NMR, IR and MS spectroscopic methods were applied for characterization and identification of all compounds. Deutrated dimethyl sulfoxide (DMSO-d_6_) was used for ^1^H NMR acquisition and TMS (tetramethyl silane) was applied as internal standard. Infrared spectroscopy was performed for all compounds using KBr disk and outstanding peaks was reported. Molecular ion peak of synthesized compounds was recorded in MS spectroscopy. Furthermore, M^+^+2 was also reported for chlorinated compounds.

### Anticholinesterase activity

According to Table 2, all compounds 4a-4l were tested against acetylcholinesterase and related IC_50_ ± SEM were calculated. Compound 4a with *ortho* chlorine substituent on phenyl ring was the most potent one in this series (IC_50_ = 0.91 ± 0.045 μM). *Para* position (compound 4c, IC_50_ = 26 ± 5 μM) was also favorable for anticholinesterase activity but on the other hands *meta* substitution of chlorine (compound 4b, IC_50_ = 85 ± 12 μM) led to the lowest inhibitory effect compared to other position for this moiety. Among compounds 4d-4f with fluorine moiety, compound 4d at position 2(*ortho*) showed the highest inhibitory property toward the acetylcholinesrase. Position 3(*meta*) was the worst position for fluorine and *para* substitution afforded an averaged potency in comparison with other positions. Compound 4g with *meta* substitution of methoxy substituent as electron donating group rendered a high potency derivative with IC_50_ = 5.5 ± 0.7 μM. A similar trend like chlorine and fluorine moieties was also observed for nitro group. Substitution of nitro group as electron withdrawing moiety at position 2(*ortho*) of the phenyl ring in compound 4i afforded the best effect. On the other hands, position 3(*meta*) was the worst position for nitro substituent (compound 4j). Insertion of nitro group at position 4(*para*) in compound 4k led to a moderate affinity for inhibition of acetylcholinesterase compared to positions 2 and 3. Compound 4l without any substituent on the phenyl ring was also one of the most potent inhibitor of acetylcholinesterase in this series. Totally, electron withdrawing moieties such as Cl, F and NO_2_ at position 2(*ortho*) of the phenyl ring can enhance the potency of synthesized phthalimide derivatives. Electron donating group like methoxy at position 3 can also increase the enzyme inhibitory effects in these series.

**Table 2:** **Results (IC**_**50**_**, μM) of *in vitro *acetylcholinesterase assay of compounds 4a-4l**

**Compound**	**4a**	**4b**	**4c**	**4d**	**4e**	**4f**	**4g**	**4h**	**4i**	**4j**	**4k**	**4l**	**Donepezil**
R	2-Cl	3-Cl	4-Cl	2-F	3-F	4-F	3-OCH_3_	4-OCH_3_	2-NO_2_	3-NO_2_	4-NO_2_	H	-
IC_50_(μM)	**0.91 ± 0.045**	85 ± 12	26 ± 5	23 ± 7	74 ± 11	42 ± 8	5.5 ± 0.7	72 ± 9	36 ± 5	59 ± 6	40 ± 4	9 ± 2	0.14 ± 0.03

Totally, electron withdrawing substituents are capable to enhance the anticholinestease activity of the phthalimide derivatives that synthesized in this research. Comparison of the different compounds with electron withdrawing moieties showed that *ortho* position is the best position for this type of substituents. Whereas, the *para* position was the worst position for all of the electron withdrawing moieties. Introducing of an electron donating group such as methoxy at *meta* position of the phenyl ring also led to the increasing of the potency. But, this change was not so effective like *ortho* substitution of chlorine atom. Absence of any moiety on the phenyl ring as observed about compound 4l could be an AChE inhibitor with an averaged potency. In fact, this compound exerted potency less than compound 4a with *ortho* chlorine as well as compound 4g with *meta* methoxy substituent.

### Molecular modeling

All ligands 4a-4l were docked into the active site of acetylcholinesterase (PDB ID: 1EVE). For performing an accurate docking procedure, the binding site of donepezil was intended as probable binding site for all ligands especially for ligand 4a. Compound 4a as the best inhibitor of AChE *in vitro* was studied *in silico* to reveal the probable binding mode of synthesized compounds perfectly (Figure 3). Superimposed state for this ligand with donepezil was also explored. According to the Figure 4, a similar binding mode and conformation as observed for donepezil, was also seen for this ligand. In fact, the structure of synthesized compounds occupies a similar region like donepezil in the active site of AChE. As seen in Figure 4, phthalimide, piperazine and benzyl rings of compound **4a** adopt a same location like indanone, piperidine and benzyl rings of the donepezil respectively.

## Conclusions

A new series of donepezil-like analogs were synthesized based on the phthalimide structure and anticholinesterase activity was assessed using Ellman test. All compounds exhibited a μM range in IC_50_ value (5.5-85 μM) for inhibition of AChE except for compound 4a that exhibited a nM range in IC_50_ value (0.91 μM). *In silico* study of compound 4a by docking method was also confirmed a similar binding mode like donepezil for this ligand.

## Competing interests

The authors declare that they have no competing interests.
